# Diversity and Population Sizes of Wintering Waterbirds in the Wetlands of the Saïss–Middle Atlas Region (North–Central Morocco): Main Survival Factors and Evaluation of Habitat Loss

**DOI:** 10.3390/ani14091352

**Published:** 2024-04-30

**Authors:** Wafae Squalli, Ismail Mansouri, Ikram Douini, Hamid Achiban, Hamza Saghrouchni, Abdelbari El Agy, Fatima Fadil, Michael Wink, Mohamed Dakki

**Affiliations:** 1 Laboratory of Functional Ecology and Environmental Engineering, Faculty of Sciences and Technology, Sidi Mohamed Ben Abdellah University (USMBA), Fez 30050, Morocco; abdelbarib@gmail.com (A.E.A.); fatimafadil66@gmail.com (F.F.); 2 Research Team “Biology, Environment and Health”, Department of Biology, Faculty of Sciences and Technologies Errachidia, Moulay Ismail University of Meknes, Errachidia 52000, Morocco; mankhori@gmail.com; 3 Laboratory of Agro-Industrial and Medical Biotechnology, Faculty of Sciences and Techniques, University Sultan Moulay Slimane, Beni Mellal 23000, Morocco; ikram.douini@gmail.com; 4 Laboratory of Geo-Environmental Analysis and Sustainable Development Planning, Sidi Mohamed Ben Abdelah University (USMBA), Fez 30050, Morocco; hamid.achiban@usmba.ac.ma; 5 Department of Biotechnology, Institute of Natural and Applied Sciences, Çukurova University, Bacalı/Sarıçam, Adana 01330, Turkey; hsaghrouchni@student.cu.edu.tr; 6 Institute of Pharmacy and Molecular Biotechnology, Heidelberg University, 69120 Heidelberg, Germany; wink@uni-heidelberg.de; 7 GREPOM/BirdLife Morocco, Résidence Oum Hani IV, Imm 22, Apt 3, Salé 11160, Morocco; dakkiisr@gmail.com

**Keywords:** wintering waterbirds, diversity, abundance, wetlands, threatening factors, natural, human-made, habitat loss, Morocco

## Abstract

**Simple Summary:**

In this paper, we present the first diagnostic of the wintering birds in the wetlands of the Saïss plain and its adjacent Atlas Mountains (north–center of Morocco). The study revealed an unexpected diversity of bird species, for which we provide the population size, with their variations, over five years (2017–2018 to 2022–2023). Among these birds, we found four remarkable species: *Oxyura leucocephala* (endangered), *Aythya ferina* (vulnerable), *Aythya nyroca* (near-threatened) and *Limosa limosa* (near-threatened). In addition, we illustrated the wetland changes during the last 20 years (2003–2023), mainly the habitat loss for wintering waterbirds.

**Abstract:**

Moroccan wetlands host up to half a million wintering birds and provide a stopover for tens of thousands of migrants, while they are inhabited by few nesting species. Most of this avifauna prefers to use the large coastal wetlands or reservoirs, while many species are dispersed across hundreds of small inland wetlands of various types. In this study, we monitored the wintering avifauna of 11 wetlands of the Saïss plain and its adjacent Atlas Mountains (north–center of Morocco), during six wintering seasons (2017–2018 to 2022–2023), with the objective of assessing the importance of this region as a waterbird wintering area. Using the richness of the species, we determine the bird population changes during this pentad and between the different types of wetlands (natural, human-made, and natural wetlands). During this study, we recorded 51 species, belonging to 17 families, among which exist four remarkable birds: the endangered *Oxyura leucocephala*, the vulnerable *Aythya ferina* and the near-threatened *Aythya nyroca* and *Limosa limosa*. Bird diversity is higher in human-made ecosystems than in peri-urban and natural ecosystems, while the populations’ size is similar in urban and non-urban wetlands. With regard to bird conservation, these inland wetlands, mainly the small ones, are threatened by recurrent droughts and various anthropic stressors, which we describe using our observations of the two last decades (2003–2023). The loss of habitat is significant, reaching 348.5 hectares, while the impacts of reduced precipitation and temperature increase are particularly evident in the mountainous natural lakes.

## 1. Introduction

In arid zones, wetlands are usually rare habitats; in North Africa, they are well known for their high productivity, in both hot and cold seasons, providing abundant foraging resources [1,2,3] to thousands of wintering and migrant waterbirds, as well as breeders [4,5]. Indeed, the majority of migratory Afropalearctic waterbirds use North African wetlands for stopover and refueling in migrations between both Africa and Europe [6,7,8,9], while nesting species are abundant but with relatively low diversity [10,11,12]. However, wetland systems are among the most seriously threatened ecosystems on the planet [13], mainly due to human influences [14,15], such as habitat backfilling, drainage, transformation and pollution. This leads to large-scale decline and redistribution of bird populations [16,17].

In Morocco, approximately 300 wetlands, including natural and artificial habitats, have been recorded in the latest national inventories [18,19]. These ecosystems are distributed from the Mediterranean coast in the north (i.e., the Nador Lagoon) to the Saharan zone in the south (i.e., Imlili and Ad-Dakhla Bay) [20,21,22,23,24,25]. Among these, 34 ecosystems or complexes were classified as Wetlands of International Importance (Ramsar sites), most of these achieving this status due to their birds [26,27].

Since 1983, a winter monitoring of waterbirds has been organized in most of the wetlands inventoried in Morocco, which reached a network of 278 sites in 2022. This monitoring revealed a large predominance, in both richness and abundance, of wintering birds in coastal lagoons, estuaries, and bays [28,29], mainly composed of waders, gulls, turns, ducks, coots, etc. [30,31]. Inland wetlands show generally low-to-medium numbers of wintering birds, most of them concentrated in artificial reservoirs, while the Middle Atlas and its northern border plain (Saïss), though rich in lakes and marshes [24,32], play a secondary role in hosting winterers; however, the increasing number of artificial reservoirs in these areas and the discovery of several remarkable birds in the small wetlands of the Saïss plain [33] show the increasing importance of these regions for wintering birds, especially for threatened species. To demonstrate this trend, we carried out waterbird monitoring between 2017–2018 and 2022–2023, in a representative sample of the wetlands of these areas. This monitoring sought to reveal the particularities of the wintering bird communities in this area, using a comparative approach between natural and artificial wetlands, with an attempt to define their eventual differences with peri-urban communities.

This study will add new information to our knowledge of the wintering avifauna of the north of Morocco. In addition, we investigated the major human and climate factors that threaten this avifauna. We undertook this investigation on the basis of a set of 11 selected sites, some of which were recently added to the wetland network covered by the International Waterbird Census program. Indeed, in the Middle Atlas, we chose three lakes that well illustrate the impact of climate change in this region, and permit us to contribute to the understanding of the major challenges facing bird conservation, knowing that these lakes are classified as Ramsar sites [18].

## 2. Materials and Methods

### 2.1. Study Area and Sites

**Figure 1:**
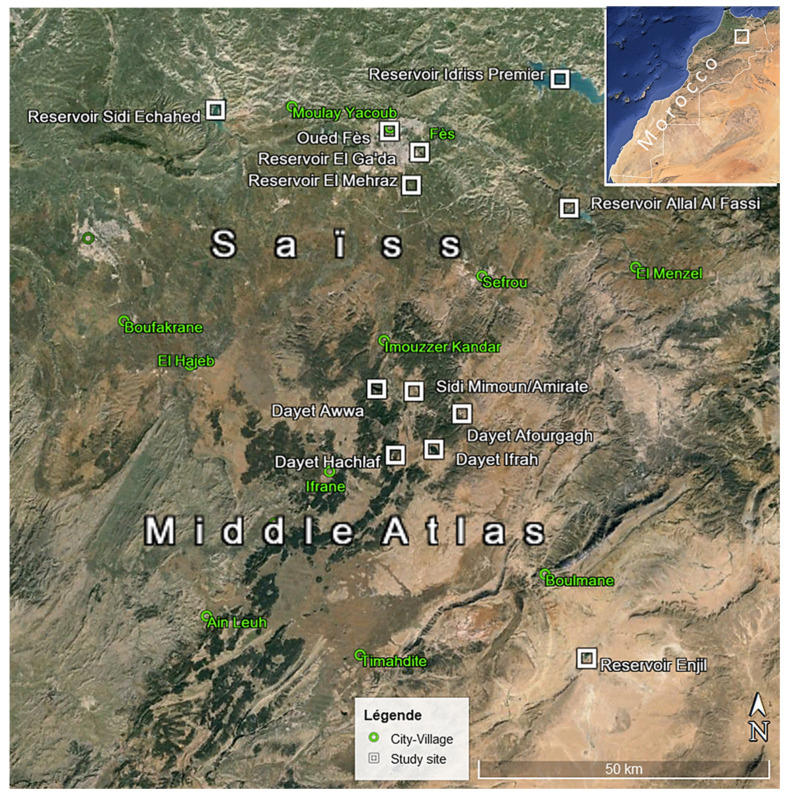
Geographical location of the study region and wetlands.

The study area, located in the north–center of Morocco (Figure 1), includes the Saïss plain in Fez region (located between 400 and 700 m above sea level) and its adjacent mountains and foothills, mainly related to Middle Atlas. This area has a Mediterranean continental climate, semi-arid to sub-humid, and is known for its hot and dry summers and humid winters [34], with great oceanic influences that increase the annual rainfall (around 500 mm in the plain and over 700 mm in mountains, with frequent snow). Most of the river systems that supply water to the Saïss plain originate in the Middle Atlas Mountains and, secondarily, in the Prérif hills.

The monitoring of wintering waterbirds was made in 11 wetlands. Five of these are in the Middle Atlas: three natural lakes (Afourgaa, Hachlaf, and Ifrah) and two small artificial reservoirs (Amirate or Sidi Mimoun and Enjil). The six others are at a lower altitude, three of these are in the Saïss plain: a river section (Oued Fès) and two small reservoirs (El Ga’da and El Mehraz) and the others are large reservoirs on the river basin of Sebou and its tributaries (Allal El Fassi, Idriss Premier and Sidi Echahed).

### 2.2. Monitoring Program

Our bird monitoring, held between 2017–2018 and 2022–2023, was focused on the winter season, but extended into late autumn and early spring (October to February), in order to cover the early arrivals and departures of winterers [6,8,35]. Each site was visited twice a month, in such a way as to cumulate 10 visits per year.

For each site, we selected fixed observation points that permit one to cover the entire waterbody. In the large reservoirs (Idriss Premier, Sidi Echahed, and Allal El Fassi), four observation points were necessary: two points being at the upstream and downstream limits of the reservoir and the two others on the sides of the reservoir, at its maximum width. In the other sites, we generally used three observation points, but in small waterbodies, a single observation point was sufficient. However, in Oued Fez, five observation points were selected along the stream banks. Each visit lasted between one and six hours.

### 2.3. Environmental Parameters

To identify the potential factors influencing the abundance of birds and their diversity, we noted, during each site visit, the hydrological state of the site, including primarily the estimated area of water and the proportion of dry and wet habitats, including the absence of water. We also delineated the studied sites in the Google Earth platform and calculated the area of the sampled habitats via polygons covering each habitat on the same satellite images (used later to calculate the densities of birds per ha). Precipitation and temperatures for the study period were obtained from the Infoclimat website (https://www.infoclimat.fr, accessed on 25 February 2024).

### 2.4. Wetland Mapping and Habitat Change Estimate

In order to determine changes over the previous two decades (2003 to 2022), the study sites were mapped using Landsat satellite series data (TM: thematic mapper, MSS: multi-spectral scanner, and OLI: operational land imager) for the winters (December) of three years (2003, 2013, and 2022). The images were downloaded from the USGS website (http://glovis.usgs.gov/, accessed on 25 February 2024) and radiometrically calibrated, corrected for geometry, and stored as 8-bit digital numbers (DNs) with UTM system.

All images were converted to radiance during the preprocessing stage (or reflectance). The following Formula (1) or (2) were used to calculate the image’s DN-to-L transform [13]. For wetlands area qualitative statistics, all images were re-projected to Lambert azimuthal equal-area projection at various periods.

Lλ = Gains ∗ DN + Bias (1)

which is also expressed as,

Lλ = (Lλmax − Lλmin)/(QCALλmax − QCALλmin) ∗ (DN − QCALλmin) + Lλmin (2)

Lλ = spectral radiance at the sensor’s aperture in mW/(cm^2^·sr·μm).

DN = digital number of the quantized calibrated pixel value.

Gains = band-specific rescaling gain factor in (mW/(cm^2^·sr·μm))/DN.

Bias = band-specific rescaling bias factor in mW/(cm^2^·sr·μm).

QCALλmax = maximum quantized calibrated pixel value in DN (corresponding to Lλmax).

QCALλmin = minimum quantized calibrated pixel value in DN (corresponding to Lλmin).

Lλmax = maximum spectral radiance that is scaled to QCALλmax.

Lλmin = minimum spectral radiance that is scaled to QCALλmin.

In addition to the analysis of wetland areas, land cover change (mainly of farmlands and urbanization) was assessed for two periods (2003–2013 and 2013–2022), by dividing their area estimated at the end of each period by the period duration.

### 2.5. Statistical Analysis

To characterize the evolution of bird assemblages in each site during the six-year study period, we used three descriptors: population size, community richness, and diversity (using the Shannon index). All statistics were realized after the test of normality with the Shapiro–Wilk test. The test showed that all studied variables follow the normal distribution. Therefore, the comparison of species and populations of wintering water birds between natural, human-made, and peri-urban wetlands, was realized with *t*-test, using only two groups per year (habitats). Further, we compared wintering species, wintering populations, and populations of threatened species with the ANOVA one way test, considering the sampling years. Moreover, the Pearson coefficient permitted us to test the correlation between wintering seasons, bird richness, and populations in order to characterize their interannual variation (effect of years on the evolution of birds).

To evaluate the principal factors impacting the wetland birds, the studied sites were subdivided into four types (n = 4 (natural, human-made, peri-urban, and non-urban)), which were considered dependent variables, while the environmental factors (n = 2 (temperature and precipitations)) and anthropogenic factors (n = 2 (farmlands and urbanization)) were considered independent variables (explanatory factors), and were analyzed with principal component analysis (PCA). The recorded results are presented in 2D and 3D plots, including axes with higher percentage of inertia and eigenvalues > 1.0. Statistics were carried out in Statgraphics Centurion XVI and significant values were considered at *p* ≤ 0.05.

## 3. Results

### 3.1. Global Diversity of Wintering Waterbirds

During the six-year winter monitoring (2017–2018 to 2022–2023) in the 11 sites, 47 waterbird species were recorded, belonging to 17 families (Table 1); among these, Anatidae and Scolopacidae are the most diversified (12 and 7 species, respectively), followed by Rallidae and Ardeidae (5 species each). This inventory represents about 44% of the regular wintering waterbirds in Morocco [36], which is quite rich for such a sample of inland waters, dominated by artificial reservoirs. The average numbers of individuals, calculated for each year, and further for the six-year monitoring, show five dominant species (*Fulica atra, Bubulcus ibis, Chroicocephalus ridibundus*, *Phalacrocorax carbo* and *Anas platyrhynchos*) that cumulate an average number of 3911 birds. On the other hand, 14 species have very low numbers and frequency; most of these are waders, generally rare in Moroccan inland waters.

Four species have a conservation status of concern, according to the IUCN Red List: *Oxyura leucocephala* (endangered), *Aythya ferina* (vulnerable), *Aythya nyroca* (near-threatened) and *Limosa limosa* (near-threatened). All others have a status of least concern. This provides a particular importance to the Middle Atlas–Saïss wetlands.

### 3.2. Interannual Variation of Bird Wintering Assemblages

**Figure 2:**
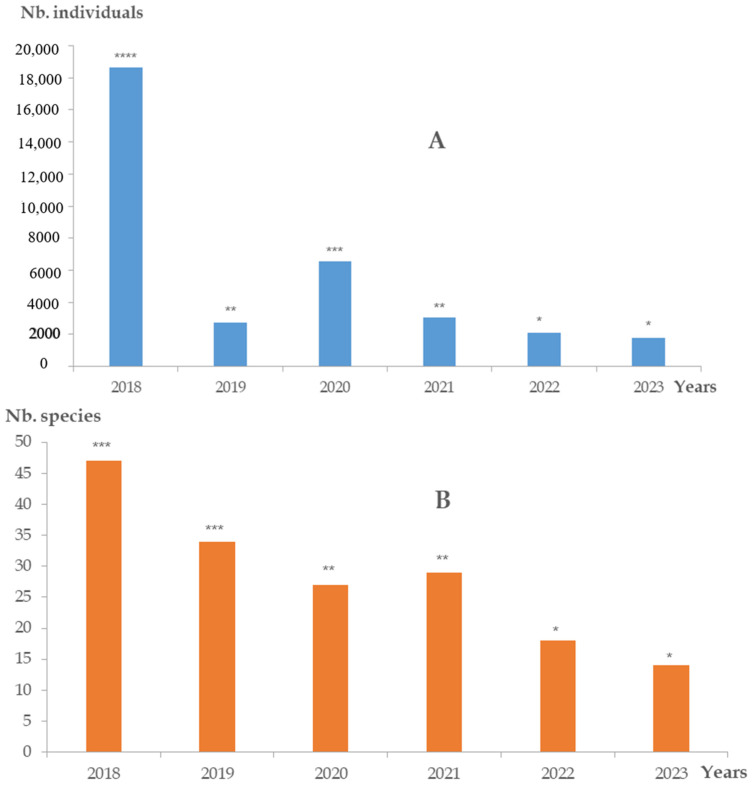
Interannual variation of wintering bird assemblages in the study area from 2018 to 2023: species richness (**A**) and total average number of individuals (**B**). * denote statistically significant differences (**** > *** > ** > *).

The interannual variation of wintering bird assemblages during the six-year monitoring (Figure 2A) shows a regular decrease of the avifauna richness (from 36 to 14 species), with the loss being 51.2%. This decline is negatively correlated with the wintering seasons (n = 4, R = −1.00, *p* < 0.001).

The total number of individuals shows a sharp decrease from 2019 (Figure 2B), but this trend is interrupted by an inversion in 2020; the lowest numbers, obtained in 2022 and 2023, are about 40% of the 2018 value. However, correlation between this decline with the richness is low (n = 4, R = 0.4000, *p* < 0.4884), probably because few dominant species govern the total abundance.

### 3.3. Comparison among Water Habitats

**Figure 3:**
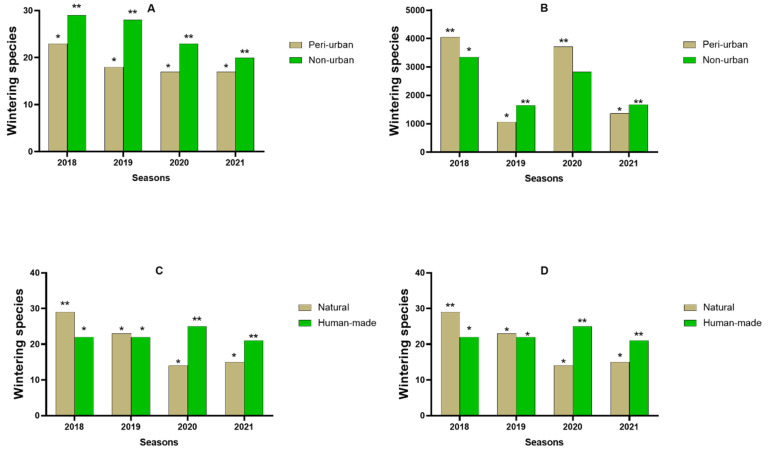
Comparison of wintering bird assemblages of peri-urban and non-urban sites, on the one hand (top diagrams), and natural and human-made sites, on the other (bottom diagrams), using their number of species profile and number of individuals profile, from 2018 to 2021. * denote statistically significant differences (** > *). (**A**): Richness of species between peri and non-urban sites; (**B**): Wintering populations between peri and non-urban sites; (**C**): Richness of species between natural and human-made sites; (**D**): Wintering populations between between natural and human-made sites.

In order to compare the wintering assemblages of the different types of sites, we used their richness and their total number of birds (Figure 3). The species richness was highest in non-urbanized aquatic ecosystems as compared with peri-urban wetlands from 2018 to 2023 (Figure 3A). In 2018, wintering populations were superior in peri-urban wetlands as compared with non-urban habitats (Figure 3B). In contrast, from 2019 to 2021, wintering populations were highest in non-urban wetlands. Meanwhile, species richness fluctuated between overwintering seasons in both natural and human-made ecosystems. In 2018, richness was highest in natural ecosystems, while in 2020 and 2021 the richness was highest in human-made wetlands. In 2019, the richness was similar in both natural and human-made systems (Figure 3C). Wintering populations were highest in human-made wetlands during 2018, 2020, and 2021, while in 2019 they were similar between both natural and human-made aquatic ecosystems (Figure 3D).

**Figure 4:**
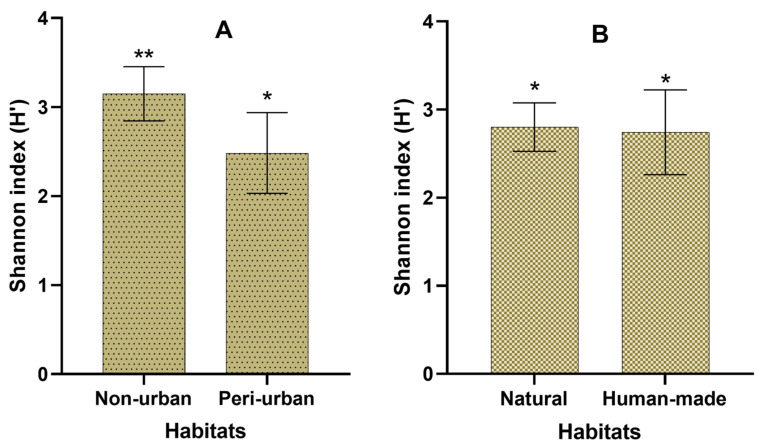
Wintering assemblage diversity (Shannon index): comparison between the peri-urban and non-urban wetlands (**A**), and between natural and human-made ecosystems (**B**). * denote statistically significant differences (** > *).

In addition, we calculated the Shannon index for the wintering bird assemblages of these types of wetlands, and compared them (Figure 4) using the sample *t*-test, which shows that, in non-urban wetlands, bird diversity is significantly higher (H’ =3.15 ± 0.30) than in peri-urban wetlands (H’ = 2.48 ± 0.45) (*p* < 0.001). The same comparison is made between natural and human-made wetlands; this reveals that their bird assemblages are statistically similar (H’ = 2.74 ± 0.48 in human-made sites and H’ = 2.8 ± 0.27 in natural wetlands).

### 3.4. Status of Species with Conservation Concern

**Figure 5:**
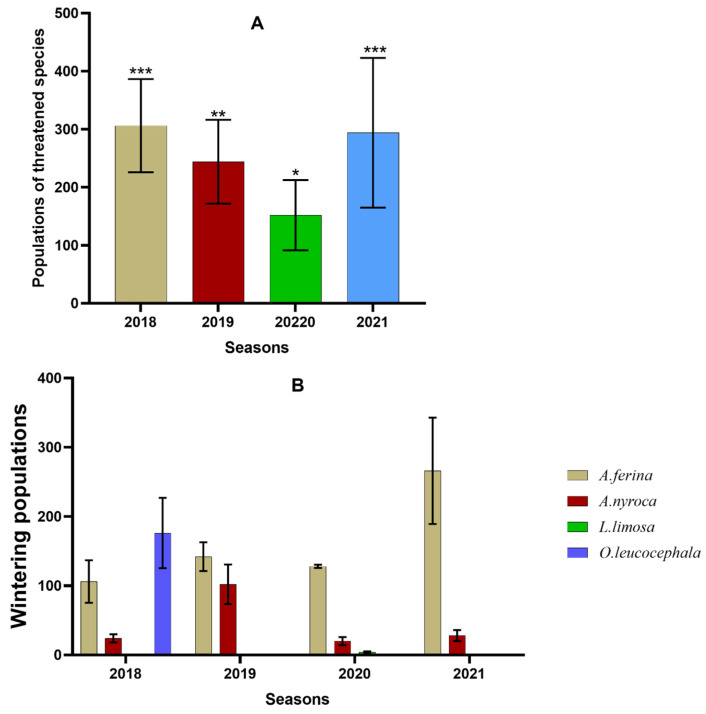
Interannual variation of the wintering abundances of the species of conservation concern (*Oxyura leucocephala, Aythya ferina, Aythya nyroca* and *Limosa limosa*) in the study sites. * denote statistically significant differences (*** > ** > *). (**A**): Total populations of threatened species; (**B**): Populations of each threatened species.

For the four species of conservation concern, we analyzed the interannual variation of their abundances during the study period (Figure 5). The variations between them were found to be significantly high in all wintering seasons (Figure 5A). Their highest wintering abundances were recorded in both the 2018 and 2021 wintering seasons, followed by those of the 2019 winter. The lowest abundances were recorded in the 2019 season, while the four species were absent in all sites during the 2022 and 2023 winters.

*Aythya ferina* was significantly abundant in the 2021 season, and its numbers slightly decreased in 2019 and 2020 (Figure 5B).

*Aythya nyroca* had its maximum numbers in 2019, then become slightly less abundant in 2020.

*Oxyura leucocephala* was recorded only in 2018, while *Limosa limosa* was observed only during winter 2020.

### 3.5. Changes in Wetlands

To understand the great variations recorded in the wintering bird communities during the monitoring period, we studied the hydrological changes in wetlands, knowing that all our study sites are located in farming areas and that some of these are also very close to urban zones (Table 2 and Table 3).

Indeed, farmlands around wetlands have expanded by 98 ha between 2003 and 2013 and by 146 ha between 2013 and 2022, while human settlements near these sites have expanded by 133 ha during 2003–2013 and 148 ha during 2013–2022.

The 11 sites, the majority of which are artificial, have been progressively created, increasing their total water surface by 404.56 ha between 2003 and 2013. However, these sites were dried on 348.5 ha from 2003 to 2013 and on 753 ha between 2013 and 2022.

In the peri-urban wetlands, the total submerged area increased by 118.96 ha during 2003–2013, while it has fallen by 169.35 ha during 2013–2022. Similarly, the water surface area of non-urban wetlands increased by 285.6 ha in 2003–2013, then fell by 583.7 ha in 2013–2022, the balance of dried water surface having reached almost 300 ha.

Natural lakes (Hachlaf, Afourgaa and Ifrah) had their submerged area increased by 306 ha during 2003–2013, but decreased by 431 ha during 2013–2022. Similarly, the area of human-made wetlands increased by 137.96 ha during 2003–2013, while it lost 246.35 ha during 2013–2022. In comparison, 125 ha of water surface was lost in natural wetlands between 2003 and 2022, compared with −108.39 ha in human-made reservoirs during the same period.

**Figure 6:**
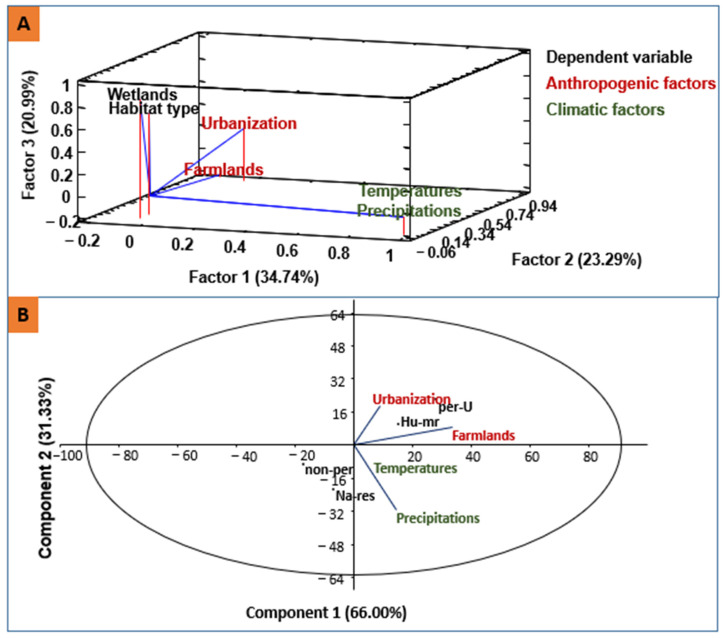
Factorial (**A**) and principal component (**B**) analysis of anthropogenic (farmland cover and urbanization) and climatic variables (temperatures and precipitation) impacting the surface areas of water bodies in studied wetlands. (Peri-U: peri-urban wetlands; Non-per: non-peri-urban wetlands; Na-res: natural reservoirs; and Hu-mr: human-made reservoirs (dams)).

**Figure 7:**
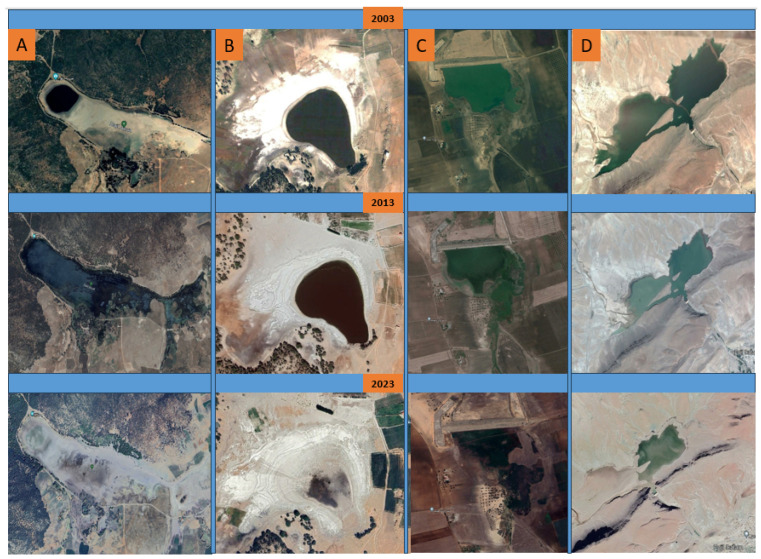
Regression and drying of the most impacted aquatic systems in the Fez-Meknes region from 2003 to 2023. (**A**) Dayt Awwaa; (**B**) Afourgaa; (**C**) El Mehraz; (**D**) Ennjil.

The factors influencing the changes of water surface area in the studied region are urbanization and farming (Figure 6), while climatic variables, mainly the annual precipitation and temperatures, do not seem directly involved in these changes. To be more accurate, increased farmlands and urbanized areas impacted principally peri-urban and human-made wetlands, compared with natural and non-peri-urban wetlands. The Middle Atlas lakes (Dayet Awwa and Afourgaa), and reservoirs of El Mehraz and Ennjil were totally dry in 2023 (Figure 7).

## 4. Discussion

Our study provides new and detailed data on the wintering waterbird populations in selected wetland habitats from central regions of Morocco, which are considered as wintering and stopover sites for Afropalearctic migratory birds. Our results define the abundance and diversity of wintering aquatic birds in peri-urban, natural, and human-made reservoirs. Furthermore, we evaluated the degradation of these arid aquatic ecosystems and their impacting factors during the last two decades, which remain one of the missing pieces of data on Moroccan wetland ecosystems.

During the six years (2018–2023) of the water-bird census, 47 wintering species were recorded in the aquatic ecosystems of the Middle Atlas–Saïss zone. This number represents 8.67% of total wintering birds in the entirety of Morocco (588 birds in total, including accidental species). These findings are relatively new for the study areas and are different from the results mentioned in continental aquatic ecosystems of Morocco. For example, bird surveys recently conducted in the reservoirs and lakes of the Midelt region (located on the south borders of the Middle Atlas) from 2015 to 2017 [33] recorded only 24 wintering bird species. In contrast, the findings mentioned in some littoral ecosystems with relatively small areas are close to our results. For example, 58 waterbird species were recorded during the 2005–2009 seasons in the Smir wetland complex located in the north of Morocco [37]. Based on a comparison between our results and those mentioned in the bibliography, we can conclude that the continental wetlands of the study area are relatively important for wintering waterbirds. This has been verified for some dominant species (*Fulica atra*), but more particularly for three ducks of conservation concern (*Oxyura leucocephala, Aythya ferina* and *Aythya nyroca*), which use both natural and human-made wetlands for wintering. However, we did not find the threatened duck *Marmaronetta angustirostris*, which has been mentioned in association with our study area from 1983 to 2019. The presence of these avian species can be explained by the abundance of water and food [38,39,40], which give these species the opportunity to breed [12].

The data on wintering birds in peri-urban and wild aquatic systems in both the northern and southern slopes of the Mediterranean basin are relatively rare and fragmentary. The majority of investigations have been concentrated on natural and coastal ecosystems compared with continental habitats [41,42,43]. In Morocco, few studies have been addressed on the wintering birds in peri-urban aquatic ecosystems [12,37,44,45]. Our former study [12] recorded 42 wintering species in rivers and reservoirs very close or inside Fez city. In non-urban wetlands, another study [33] recorded only 24 wintering and migrant birds in peri-urban ecosystems, compared with 91 species in non-urban aquatic ecosystems (reservoirs, rivers, and lakes) in the Midelt region, which is 180 km away from the Saïss region. A similar study undertaken in southern Tunisia [42], in the Saharan wetlands of Douz, found only 34 wintering bird species, dominated by wading birds. Bird monitoring undertaken in Algeria [46], in wetlands of the central zones, including reservoirs, lakes and rivers, revealed 52 waterbird species, among which only 22 wintering species.

The analysis of our findings and of those of the literature confirms the medium richness of wintering birds in non-urbanized ecosystems compared with peri-urban habitats. Despite the lack of data on factors governing the low avian diversity in North African peri-urbanized habitats, few ornithologists have mentioned disturbances as a major impact of urbanization [12,18,32]; however, more factors have been documented in the peri-urban aquatic habitats of Fez [12], including water pollution, fishing activities, and presence of visitors.

In this study, despite the lower diversity of wintering birds in the urbanized systems, these systems host important populations of globally threatened species, such as *Oxyura leucocephala* (up to 88 individuals), which was formerly reported from this region as having more than 500 wintering individuals (unpublished data obtained from the International Waterbird Census database). This species has greater numbers in other peri-urban wetlands, as the Moroccan littoral marshlands of Fouwarate and Sidi Boughaba, located near Kenitra and Mehdiya cities, respectively [6,47]; the great lake of Tonga near El Kala city in northwest Algeria [48]; and in Cullera wetland near Valencia in Spain [49,50].

The human-made reservoir that are the subjects of this study also hosted higher bird wintering numbers, in comparison with natural ecosystems. This indicates the favorable roles of this human-made infrastructure for wintering and migrating birds, as well as for resident breeding populations. This finding contrasts with studies that indicate the negative effect of dams on avian species and their natural habitats, more especially a decrease in food abundance and lower autumn water levels [51,52]. In our case, the lowering of the water level is not specific to artificial wetlands, as natural lakes (Awwaa, Afourgaa, and Ifrah) were completely dry for between 2 and 4 successive years. Their birds move elsewhere, including to the Middle Atlas lakes; however, to clarify these displacements, we need more studies of the connectivity aspects in the region, even in the north of Morocco.

Using GIS tools, we have demonstrated that the wetlands of the study area have lost 348.5 ha because of anthropogenic and natural factors. This issue is accentuated in Morocco, such as in the case of the Loukkos and Gharb wetlands (northwest Morocco) which have lost 34–93% of their areas between 1912 and 2020 [53], in Tunisia and Algeria [54,55]. In our case, the increased surfaces of farmlands and urbanized zones decreased the area of water bodies, principally in the peri-urban and human-made wetlands, compared with natural and non-peri-urban wetlands. In contrast, the impacts of climatic variables, including annual precipitations and temperature, affected mountain natural lakes, but can also affect littoral wetlands [56,57]. Currently, climate change, mainly drought crises, constitute the major factor of deterioration of the Middle Atlas wetlands [32,58], as well as in the remainder of Morocco. Indeed, the temperature increase, expressed by the number of hot days or warm months, is very significant [59], while the number of cold nights and precipitation days are gradually decreasing, drying out several wetlands [60]. This is due to both the rarity of the water input and evapotranspiration [61,62] and affects habitat integrity [63] and water substrate [64].

We assume that these factors can explain the reduction of wintering birds recorded in our study [65]. Indeed, in 2022 and 2023, the threatened species were absent from our study sites, more precisely from El Mehraz reservoir and the Middle Atlas lakes [12].

## 5. Conclusions

In summary, our monitoring of the wintering avifauna in the wetlands of Saïss–Middle Atlas region revealed some important wintering populations, including those of four globally threatened species. Inside this region, the comparison between communities of these sites, using species richness, showed significant differences between natural, peri-urban, and human-made aquatic ecosystems. Equally, we estimated the great variation, and more precisely decrease, of wetlands areas due to natural and anthropogenic factors. Besides their prospective importance for a possible future large-scale comparative investigation of biological diversity in other north African and Mediterranean zones, these results could be of great interest for the employment of future long-term monitoring and conservation measures, mainly to protect aquatic waterbirds, more particularly the endangered species in the arid environment.

## Figures and Tables

**Table 1:** Inventory of the wintering waterbirds recorded in the 11 study sites during the six-year monitoring (2017 to 2023), with their total number of individuals. phenological status (RB: resident breeders, WV: winter visitor, PM: passage migrant, OB: occasional breeder, occasional winter occurrence of regular migrant species—BM, PM) and IUCN conservation status (EN: endangered, LC: least concern, NT: near threatened) of recorded species. * Average of the 6 winter abundances, each winter value being an average of 2 censuses.

Families\Species	Common Name	PhenologicalStatus in Morocco	IUCN Status	Average Number of Individuals *									
				2018	2019	2020	2021	2022	2023	Total	Average	Min.	Max.
**Accipitridae**													
*Circus aeruginosus*	Western marsh Harrier	RB, WV, PM	LC	35	15	10	5			65	11	5	35
**Pandionidae**													
*Pandion haliaetus*	Osprey	PM, WV, RB	LC	9	11		2	7		29	5	2	11
**Anatidae**													
*Anas acuta*	Northern Pintail	WV, PM, OB	LC	44	11	11				66	11	11	44
*Spatula clypeata*	Northern Shoveler	WV, PM, OB	LC	554	34	162	174	20	22	966	161	20	554
*Anas crecca*	Eurasian teal	WV, PM	LC	182	70		48			300	50	48	182
*Anas platyrhynchos*	Mallard Duck	RB, WV	LC	1243	221	481	314	140	120	2519	420	120	1243
*Aythya ferina*	Common Pochard	WV, PM, OB	**VU**	321	71	64	133			589	99	64	321
*Aythya nyroca*	Ferruginous Duck	RB, WV	**NT**	88	51	10	14			163	28	10	88
*Mareca strepera*	Gadwall	WV, OB	LC	206	44					250	42	44	206
*Netta rufina*	Red-crested Pochard	RB, WV	LC	2			2			4	1	2	2
*Oxyura leucocephala*	White-headed Duck	WV, RB, PM	**EN**	88						88	15	88	88
*Spatula querquedula*	Garganey	PM, OW	LC	4						4	1	4	4
*Tadorna ferruginea*	Ruddy Shelduck	RB	LC	690	119	91	410			1310	219	91	690
*Tadorna tadorna*	Common Shelduck	WV	LC	82			2			84	14	2	82
**Charadriidae**													
*Charadrius alexandrinus*	Kentish Plover	RB, PM, WV	LC	1	1					2	1	1	1
*Charadrius dubius*	Little ringed Plover	RB, PM, WV	LC	114	1					115	20	1	114
*Charadrius hiaticula*	Common ringed Plover	PM, WV	LC	320	3	294	20	20		657	110	3	320
**Glareolidae**													
*Glareola pratincola*	Collared Pratincole	BM, PM, OW	LC	2	2					4	1	2	2
**Laridae**													
*Chroicocephalus ridibundus*	Black-headed Gull	WV, PM, OB	0	2793	328	45	500	650	450	4766	795	45	2793
**Recurvirostridae**													
*Himantopus himantopus*	Black-winged Stilt	BM/RB, PM, WV	LC	317	5	90	86	120	70	688	115	5	317
*Recurvirostra avosetta*	Pied Avocet	PM, WV, OB	LC	44		2	4			50	9	2	44
**Scolopacidae**													
*Actitis* *hypoleucos*	Common Sandpiper	PM, WV	LC	60	35		8			103	18	8	60
*Calidris minuta*	Little Stint	PM, WV	LC	1	1					2	1	1	1
*Gallinago gallinago*	Common Snipe	WV, PM	LC	2	2					4	1	2	2
*Limosa limosa*	Black-tailed Godwit	PM, WV	**NT**	2		2				4	1	2	2
*Scolopax rusticola*	Eurasian Woodcock	WV	LC	1						1	1	1	1
*Tringa nebularia*	Common Greenshank	PM, WV	LC	11						11	2	11	11
*Tringa ochropus*	Green Sandpiper	PM, WV	LC	2	1		2	2		7	2	1	2
**Ciconiidae**													
*Ciconia ciconia*	White Stork	PM, BM, WV	LC	508	262	6			20	796	133	6	508
**Alcedinidae**													
*Alcedo atthis*	Common Kingfisher	RB, WV	LC	3		2				5	1	2	3
*Fulica atra*	Eurasian Coot	RB, WV	LC	4012	860	991	540	360	430	7193	1199	360	4012
*Fulica cristata*	Red-knobbed Coot	RB	LC	566	208	200	150	150		1274	213	150	566
**Rallidae**													
*Gallinula chloropus*	Common Moorhen	RB, WV	LC	121	53	36	24	24	80	338	57	24	121
*Porphyrio porphyrio*	Western Swamphen	RB	LC	14	9	4				27	5	4	14
*Rallus aquaticus*	Water Rail	WV, RB	LC	1	1					2	1	1	1
**Ardeidae**													
*Ardea alba*	Great Egret	WV, PM	LC	5		1	1	1		8	2	1	5
*Ardea cinerea*	Grey Heron	PM, WV, OB	LC	105	24	38	27	26	14	234	39	14	105
*Ardeola ralloides*	Squacco Heron	BM, PM, OW	LC						4	4	1	4	4
*Bubulcus ibis*	Cattle Egret	RB, PM, WV	LC	3130	17	2500	130	150	120	6047	1008	17	3130
*Egretta garzetta*	Little Egret	RB, PM, WV	LC	333	2	210	70	90	150	855	143	2	333
**Threskiornithidae**													
*Platalea leucorodia*	Eurasian Spoonbill	PM, WV, RB	LC	296	115	94	50			555	93	50	296
*Plegadis falcinellus*	Glossy Ibis	PM, WV, OB	LC	4			4	4		12	2	4	4
**Phoenicopteridae**													
*Phoenicopterus roseus*	Greater Flamingo	PM, WV, FB	LC	26			4			30	5	4	26
**Podicipedidae**													
*Podiceps cristatus*	Great crested Grebe	RB, WV	LC	452	6	440		60	50	1008	168	6	452
*Podiceps nigricollis*	Black-necked Grebe	WV, RB	LC	71	8	11	14			104	18	8	71
*Tachybaptus ruficollis*	Little Grebe	RB, WV	LC	260	20	99	71	75	45	570	95	20	260
**Phalacrocoracidae**													
*Phalacrocorax carbo*	Great Cormorant	RB, WV	LC	1509	115	651	238	188	230	2931	489	115	1509
	**Total individuals**			18,634	2726	6545	3047	2087	1805	34,844	5826	1805	18,634
	**Nb. species**			47	34	27	29	18	14			14	47

**Table 2:** Status, nature, and location of the study sites in Fez–Meknes region. * Ram: Ramsar site; SIBE: Site of Biological and Ecological Interest; NCS: no conservation status.

Sites	Type	Date of Creation	Location	Conservation Status *	Drying Period
Human-made sites					
El Gâada	Reservoir	1991	Peri-urban	NCS	
El Mehraz	Reservoir	1992	Non-urban	NCS	2020–2023
Allal El Fassi	Reservoir	1990	Non-urban	NCS	
Idriss Premier	Reservoir	1973	Non-urban	SIBE	
Ennjil	Reservoir	1995	Non-urban	NCS	
Sidi Echahed	Reservoir	1996	Non-urban	NCS	
Sidi Mimoun (Amirate)	Reservoir	< 1980	Non-urban	NCS	
Natural sites					
Oued Fez	River		Peri-urban	NCS	
Dayet Afourgaa	Lake/marshland		Non-urban	NCS	2022–2023
Dayet Hachlaf	Lake/marshland		Non-urban	Ram and SIBE	
Dayet Ifrah	Lake		Non-urban	Ram and SIBE	

**Table 3:** Comparison of wetlands’ habitat loss and evolution of land cover around studied sites from 2003 to 2022 (positive values indicate the increase of areas while negative values indicate the decrease of covered areas over time).

Parameter	Surface Water Area Loss/Gain (ha)	Periods	Estimated Difference	T	df	*p*
Total submerged area of sites	+405	2003–2013	+17.275	−1.723	10	0.116
	−348	2003–2022	−69.619	1.248	10	0.241
	−753	2013–2022	−133.687	3.764	10	0.003
Farmlands	+98	2003–2013	+12.116	−0.944	10	0.367
	+244	2003–2022	+20.815	−1.149	10	0.277
	+146	2013–2022	+9.338	−1.307	10	0.220
Urbanization	+133	2003–2013	+5.894	−1.497	10	0.165
	+281	2003–2022	+6.902	−1.754	10	0.109
	+148	2013–2022	+3.141	−1.806	10	0.101

## Data Availability

The datasets used and/or analyzed during the current study are available from the corresponding author on reasonable request.
